# Effects of Adoptive Transfer of Tolerogenic Dendritic Cells on Allograft Survival in Organ Transplantation Models: An Overview of Systematic Reviews

**DOI:** 10.1155/2016/5730674

**Published:** 2016-07-28

**Authors:** Yanni Zhou, Juan Shan, Yingjia Guo, Shengfu Li, Dan Long, Youping Li, Li Feng

**Affiliations:** ^1^Key Laboratory of Transplant Engineering and Immunology of The Ministry of Health, Regenerative Medical Research Centre, West China Hospital, Sichuan University, Chengdu 610041, China; ^2^Chinese Cochrane Centre, Chinese Evidence-Based Medicine Centre, West China Hospital, Sichuan University, Chengdu 610041, China

## Abstract

*Objective*. To dissect the efficacy of Tol-DC therapy with or without IS in multiple animal models of transplantation.* Methods and Results*. PubMed, Medline, Embase, and the Cochrane Library were searched for reviews published up to April 2015. Six systematic reviews and a total of 61 articles were finally included. Data were grouped by organ transplantation models and applied to meta-analysis. Our meta-analysis shows that Tol-DC therapy successfully prolonged allograft survival to varying extents in all except the islet transplantation models and with IS drugs further prolonged the survival of heart, skin, and islet allografts in mice, but not of heart allografts in rats. Compared with IS drugs alone, Tol-DC therapy with IS extended islet allograft survival in rats but failed to influence the survival of skin, small intestine, and heart allografts in rats or of heart and skin allografts in mice. *Conclusion*. Tol-DC therapy significantly prolonged multiple allograft survival and further prolonged survival with IS. However, standardized protocols for modification of Tol-DC should be established before its application in clinic.

## 1. Introduction

Transplantation is one of the most effective methods of extending life for patients with end-stage organ failure. However, the immunosuppressive (IS) agents commonly used to prevent graft-versus-host disease and host-versus-graft disease compromise the recipient's immune system and are associated with side effects such as infection and recurrence of disease, thus decreasing the patient's quality of life. For this reason, induction of donor-specific tolerance without impairment of immune defense remains the holy grail of transplantation research.

Dendritic cells (DCs), first described in 1973 [1], are the most effective antigen-presenting cells and are key regulators of a balanced immune system by virtue of their dual immunogenic and tolerogenic functions. Immunogenic DCs have been developed as positive therapeutic vaccines to elicit antitumor responses. The first DC vaccine, sipuleucel-T (PROVENGE®), was approved by the FDA in 2010 and has since been successfully used in prostate cancer treatment [2]. In contrast, tolerogenic DCs (Tol-DCs) lack essential costimulatory signals and/or express inhibitory signals and play a role in tolerance induction. Evidence indicates that Tol-DCs have great therapeutic potential in autoimmunity and allergy [3]. To date, several phase I trials assessing safety of Tol-DCs in rheumatoid arthritis and refractory Crohn's disease patients were conducted [4, 5]. Moreover, mounting evidence shows that Tol-DCs are able to induce donor-specific T cell hyporesponsiveness and prolong allograft survival. As such, negative vaccines based on Tol-DCs have great potential to prevent transplant rejection. The safety of autologous Tol-DCs has so far been demonstrated in type I diabetes patients [6] and is currently being tested by Moreau et al. in kidney transplant recipients [7]. However, whether Tol-DCs can effectively prolong allograft survival and show superiority to other forms of IS therapy remains controversial. Here, we present the results of a meta-analysis of the efficacy of Tol-DCs in multiple animal models of transplantation. We evaluated allograft survival time after treatment with Tol-DCs alone, compared the relative superiority of single therapy with Tol-DCs or IS, and looked for evidence of synergy between Tol-DC and IS therapy in promoting allograft survival.

## 2. Methods

### 2.1. Criteria for Considering Reviews for Inclusion and Exclusion

We included systematic reviews that focused on the effects of Tol-DC injection on allograft survival compared with untreated groups in any kind of transplantation model. To be included, the reviews had to describe the outcome of interest.

### 2.2. Search Methods for Identification of Reviews

Comprehensive literature searches were conducted in PubMed, Medline, Embase, and the Cochrane Library from database inception until April 2015. We identified relevant systematic reviews using the following as MeSH or text words: “transplantation,” “dendritic cells,” “tolerance,” and “review.” To ensure comprehensive and up-to-date coverage of the evidence base and to make recommendations for future reviews, we also searched for and considered primary articles that were potentially eligible for, but not yet included in, published reviews.

### 2.3. Selection of Reviews and Articles

We screened reviews according to the inclusion criteria above and also included new primary studies, excluding duplicates and those already included in the reviews. For primary articles, we included only those that provided data applicable to meta-analysis on (i) Tol-DCs versus untreated and/or (ii) Tol-DCs in combination with IS agents (including immunosuppressive drugs and/or costimulatory blockers) versus Tol-DCs alone and (iii) Tol-DCs in combination with IS agents versus IS alone. We also excluded studies that were included in the reviews but did not provide data applicable to meta-analysis.

### 2.4. Data Extraction

For the eligible reviews, two reviewers independently extracted information on author name, publication year, transplantation model, outcomes measured, whether a meta-analysis was conducted, and quality assessment of the original articles. For primary articles, information was extracted on transplantation model, interventions, group comparisons, and outcomes measured. Disagreements were resolved by consensus.

### 2.5. Quality Assessment of Systematic Reviews

The methodological quality of the included systematic reviews was appraised by two independent reviewers using the Assessment of Multiple Systematic Reviews (AMSTAR) tool [9]. AMSTAR consists of 11 questions, each with “Yes,” “No,” “Can't Answer,” or “Not Applicable” answers, and checks for the following items: (1) “a priori” study design; (2) duplicate reviewers for study selection and data extraction; (3) comprehensive literature search; (4) publication status as an inclusion criterion (i.e., gray or unpublished literature); (5) list of studies included/excluded; (6) characteristics of the included studies; (7) scientific quality assessment and documentation; (8) appropriate formulation of conclusions (based on methodological rigor and scientific quality of the studies); (9) appropriate methods of combining studies (homogeneity test, effects model, and sensitivity analysis); (10) assessment of publication bias (graphic and/or statistical test); and (11) inclusion of conflict of interest statement. Disagreements were resolved by consensus.

### 2.6. Data Synthesis

Data were divided into six groups according to the transplantation model and then further divided into subgroups based on animal species. For each model, we grouped the data by intervention as follows: Tol-DCs versus untreated, Tol-DCs in combination with IS versus Tol-DCs, and Tol-DCs in combination with IS versus IS. The primary end point of our meta-analysis was allograft survival time. For each study, we calculated the summary mean difference and 95% confidence intervals (CI) for the end point. We pooled studies using a random effects model, making the assumption that individual studies estimated different treatment effects. We examined heterogeneity in the main analysis and subgroup analysis by *Q* statistic and *I* ^2^ index. Three articles were excluded from our summary table (Table 3) and discussion because they contained only a single set of data and the evidence was too weak to be included [16–18]. However, data from those articles are mentioned individually in the Results.

### 2.7. Ethics

No ethical approval was required.

## 3. Results

### 3.1. Results of Search and Selection

**Figure 1:**
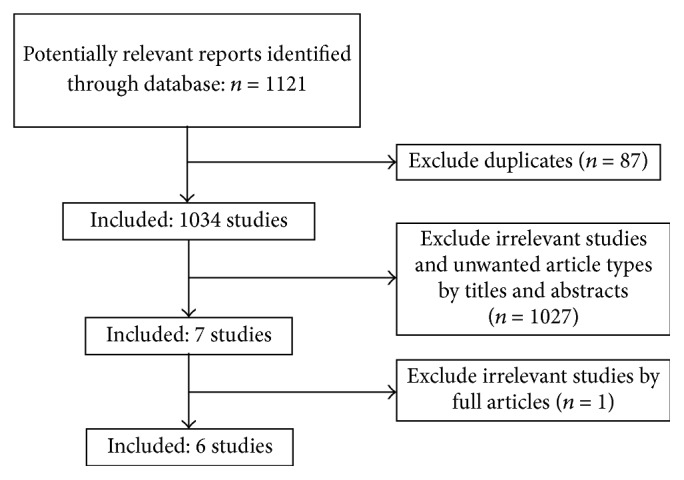
Flow diagram of searching and selection for included systematic reviews.

Our research identified 1121 reports, of which 87 were excluded as duplicates. Screening by the titles and abstracts, we excluded 1027 articles for irrelevant themes or unwanted article types and 7 were selected to be read in their entirety. Of those, 1 systematic review was excluded for irrelevant theme and 6 systematic reviews assessing the efficacy of Tol-DC treatment in animal models of heart, liver, kidney, small intestine, skin, and islet transplantation satisfied our inclusion and exclusion criteria and were further evaluated (Figure 1) [10–15]. Of the 112 studies included in the six systematic reviews, 65 studies were excluded because of inadequate data for meta-analysis (heart 28, skin 16, kidney 9, islet 8, small intestine 3, and liver 2), and the remaining 47 studies were included in our overview [8, 16, 17, 19–63]. We also included 14 newly identified primary articles (heart 8 [64–71], skin 3 [8, 71, 72], and islet 3 [18, 73, 74]). Thus, we evaluated a total of 61 studies (Table 1).

### 3.2. Description of Included Reviews

Of the six included reviews, which were published between 2012 and 2014, only one conducted a meta-analysis [10–15]. The remaining five had incomplete information, such as omission of sample size or standard deviation, and applied semiquantitative methods to analyze the collected data. The kidney and islet transplantation studies included both mouse and rat models, whereas the skin and heart studies included only mouse models and the small intestine and liver studies included only rat models (Table 1). Studies using either model were eligible for our overview.

### 3.3. Methodological Quality of Included Reviews

We assessed the methodological quality of the six included reviews using AMSTAR. The scores ranged from 5 to 8, with points deducted for Item 4 (status of the publication as an inclusion criterion), Item 5 (list of studies included/excluded), Item 9 (appropriate methods of combining studies), and Item 10 (assessment of publication bias) (Table 2). Although the systematic reviews are of only moderate to high quality, it should be borne in mind that there are no conventional criteria for quality assessment of animal studies and no clinical data are available for an equivalent analysis of humans.

### 3.4. Effects of Interventions on the Survival of Organ Allografts

#### 3.4.1. Liver Transplantation Models

In rats, infusion of Tol-DCs promoted liver allograft survival for an additional 18 days compared with no treatment (mean and 95% CI; 18.17, 11.02 to 25.33) (Figure 3). One study (excluded from the overall evaluation) reported that Tol-DC + IS therapy was more effective in prolonging graft survival than either Tol-DCs or IS alone (mean ± SD, 112 days ± 19.0 versus 58 ± 3.7 versus 54 ± 2.4, resp.) [16].

#### 3.4.2. Renal Transplantation Models

Tol-DC therapy prolonged renal graft survival by 17 days in rats (17.72, 13.35 to 22.10) (Figure 3). Moreover, one study reported that Tol-DCs + IS extended graft survival significantly longer than Tol-DCs or IS alone (38.7 days ± 40.0 versus 5.0 ± 2.2 versus 7.5 ± 1.2, resp.) [17].

#### 3.4.3. Heart Transplantation Models

**Figure 2:**
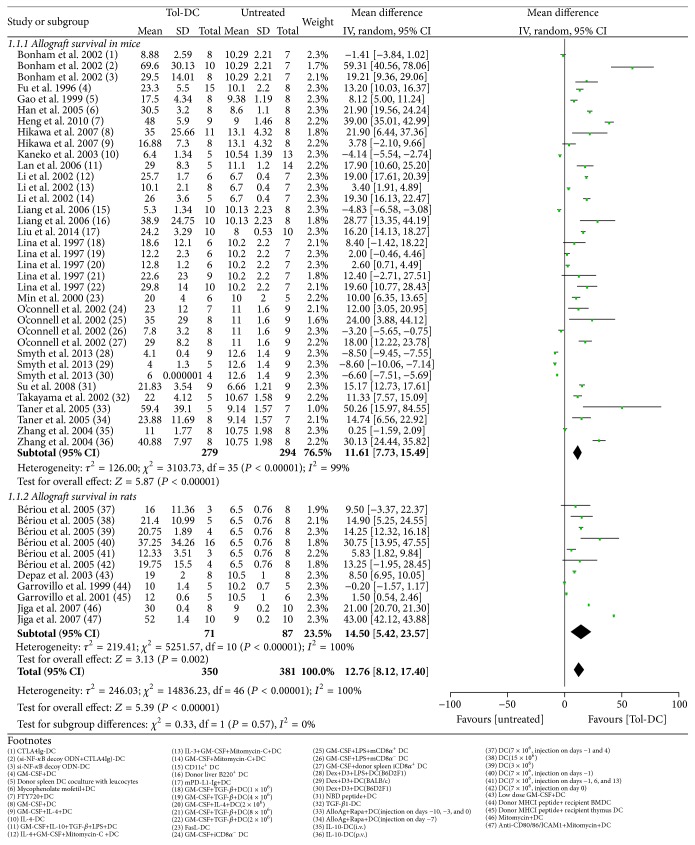
Mean difference (95% confidence intervals) for Tol-DC infusion versus untreated groups for allografts survival in heart. All studies involved used MHC complete mismatch models either in rats or in mice. In the footnotes, “−” before “DC” means genetic modification and “+” means substances added in the culture medium. If not specialized, DC refers to donor bone borrow DC; when injection time is involved, we take the transplantation day as day 0. i.v.: intravenous; p.v.: portal vein.

Heart grafts survived 14 days longer in Tol-DC-infused rats than in untreated rats (14.21, 6.11 to 22.31) (Figure 2). However, Tol-DC + IS therapy failed to further prolong allograft survival compared with Tol-DCs alone (60.21 days, −43.78 to 164.20) (Figure 4) or IS alone (57.56 days, −59.15 to 174.27) (Figure 5). In mice, infusion of Tol-DCs extended graft survival by 11 days (11.61, 7.73 to 15.49) (Figure 2). Tol-DC + IS therapy extended graft survival compared with Tol-DCs alone (5.05 days, 1.53 to 8.57) (Figure 4) but not with IS alone (1.72 days, −3.67 to 7.10) (Figure 5).

#### 3.4.4. Small Intestine Transplantation Models

**Figure 3:**
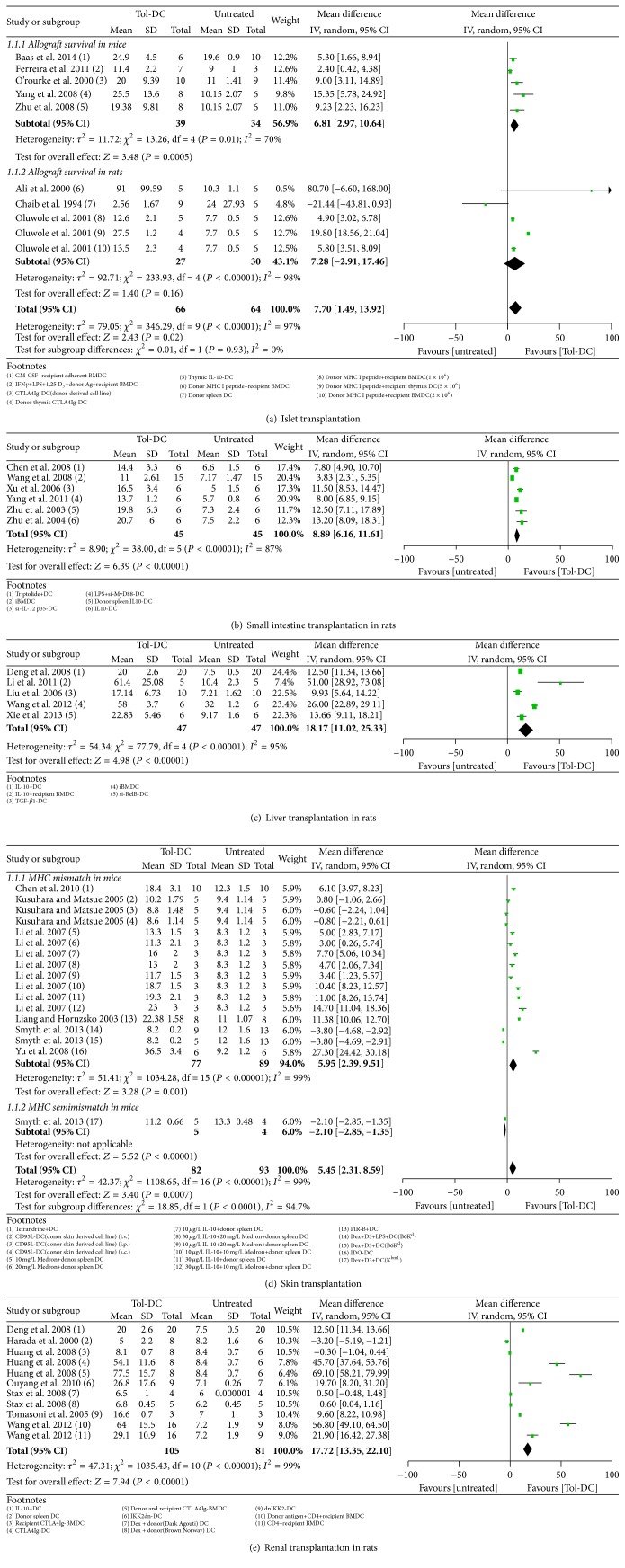
Mean difference (95% confidence intervals) for Tol-DC infusion versus untreated groups for allografts survival in islet (a), small intestine (b), liver (c), skin (d), and renal (e) transplantation models. Except for MHC semimismatch model in skin transplantation group, all other studies involved used MHC complete mismatch models either in rats or in mice. In the footnotes, “−” before “DC” means genetic modification and “+” means substances added in the culture medium. If not specialized, DC refers to donor bone borrow DC; when injection time is involved, we take the transplantation day as day 0. i.v.: intravenous; i.p.: intraperitoneal; s.c.: subcutaneous.

In rats, Tol-DC therapy prolonged graft survival by 8 days (8.89, 6.16 to 11.61) (Figure 3); however, Tol-DC + IS therapy failed to promote graft survival longer than IS therapy alone (8.97 days, −3.75 to 21.07) (Figure 5).

#### 3.4.5. Islet Transplantation Models

**Figure 4:**
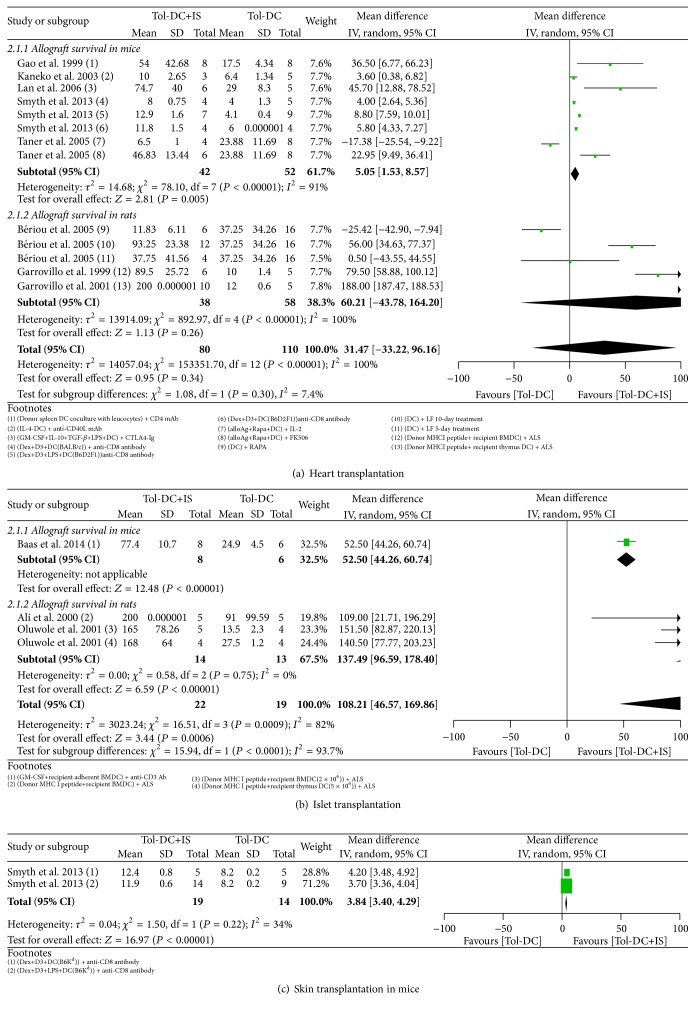
Mean difference (95% confidence intervals) for Tol-DC in combination with IS versus Tol-DC alone for allografts survival in heart (a), islet (b), and skin (c) transplantation models. In the footnotes, “−” before “DC” means genetic modification and “+” inside the parentheses means substances added in the culture medium and “+” outside the parentheses means combined IS agents. If not specialized, DC refers to donor bone borrow DC.

**Figure 5:**
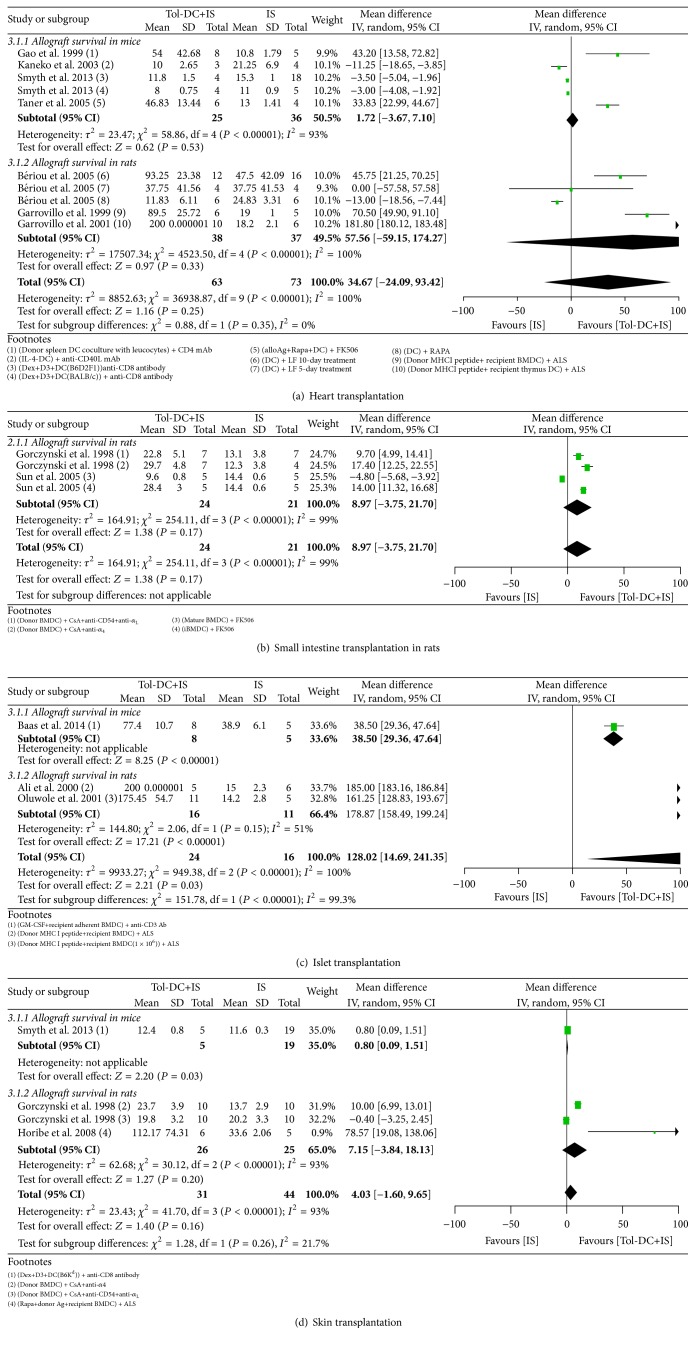
Mean difference (95% confidence intervals) for Tol-DC in combination with IS versus IS alone for allografts survival in heart (a), small intestine (b), islet (c), and skin (d) transplantation models. Rats used in study Gorczynski 1998 [8] in small intestine and skin models were MHC semimismatch. In the footnotes, “−” before “DC” means genetic modification and “+” inside the parentheses means substances added in the culture medium and “+” outside the parentheses means combined IS agents. If not specialized, DC refers to donor bone borrow DC.

Infusion of Tol-DCs failed to prolong allograft survival in rats (7.28 days, −2.91 to 17.46) (Figure 3). However, Tol-DC + IS therapy was significantly better than Tol-DCs or IS alone in prolonging graft survival (137.49 days, 96.59 to 178.40 and 177.83 days, 160.05 to 195.62, resp.) (Figures 4 and 5). In mice, Tol-DC therapy prolonged allograft survival by 6 days (6.81, 2.97 to 10.64) (Figure 3). One included study reported that Tol-DCs + IS facilitated graft survival for significantly longer than Tol-DCs or IS alone (77.4 days ± 10.7 versus 24.9 ± 4.5 versus 38.9 ± 6.1, resp.) [18].

#### 3.4.6. Skin Transplantation Models

The systematic reviews did not include studies of the effects of Tol-DC therapy alone on skin allograft survival in rats. Nevertheless, our analysis indicates that graft survival was no better in rats treated with Tol-DC + IS therapy than with IS alone (7.15 days, −3.84 to 18.13) (Figure 5). In mice, Tol-DC therapy prolonged graft survival by 5 days (5.45, 2.31 to 8.59) (Figure 3), and Tol-DCs + IS had a significantly better outcome compared with Tol-DCs alone (3.84 days, 3.40 to 4.29) (Figure 4) but not with IS alone (0.45 days, 0.00 to 0.89) (Figure 5).

### 3.5. Effects of Different Interventions on Allograft Survival

#### 3.5.1. Tol-DCs versus No Treatment

Tol-DC therapy prolonged allograft survival in all transplantation models in rats and/or mice, with the exception of the islet transplantation model in rats. Ranked in order from longest to shortest allograft survival time, Tol-DC therapy was most efficacious for liver, kidney, heart, small intestine, and islet allografts in rats and heart, islet, and skin allografts in mice (Table 3, Figures 2 and 3).

#### 3.5.2. Tol-DCs + IS versus Tol-DCs Alone

In rats, Tol-DCs + IS further prolonged the survival of islet allografts (137.49 days, 96.59 to 178.40), but not heart allografts (60.21 days, −43.78 to 164.20), compared with Tol-DCs alone. In mice, Tol-DCs + IS were superior to Tol-DCs alone in prolonging survival of both heart and skin allografts (5.05 days, 1.53 to 8.57 versus 3.84 days, 3.40 to 4.29, resp.) (Table 3 and Figure 4). Three studies of mouse islet [18], rat liver [16], and rat kidney [17] transplantation models reported better outcomes with Tol-DCs + IS than with Tol-DCs alone.

#### 3.5.3. Tol-DCs + IS versus IS Alone

In rats, Tol-DCs + IS led to better outcomes than IS alone in the survival of islet allografts, but not of heart, skin, or small intestine allografts, whereas in mice, Tol-DCs + IS were not significantly better than IS alone in prolonging the survival of heart or skin allografts (Table 3 and Figure 5). In three studies, the survival of mouse islets, rat liver, and rat kidneys was extended for significantly longer with Tol-DCs + IS than with IS alone [16–18].

#### 3.5.4. Subgroup Analysis

**Figure 6:**
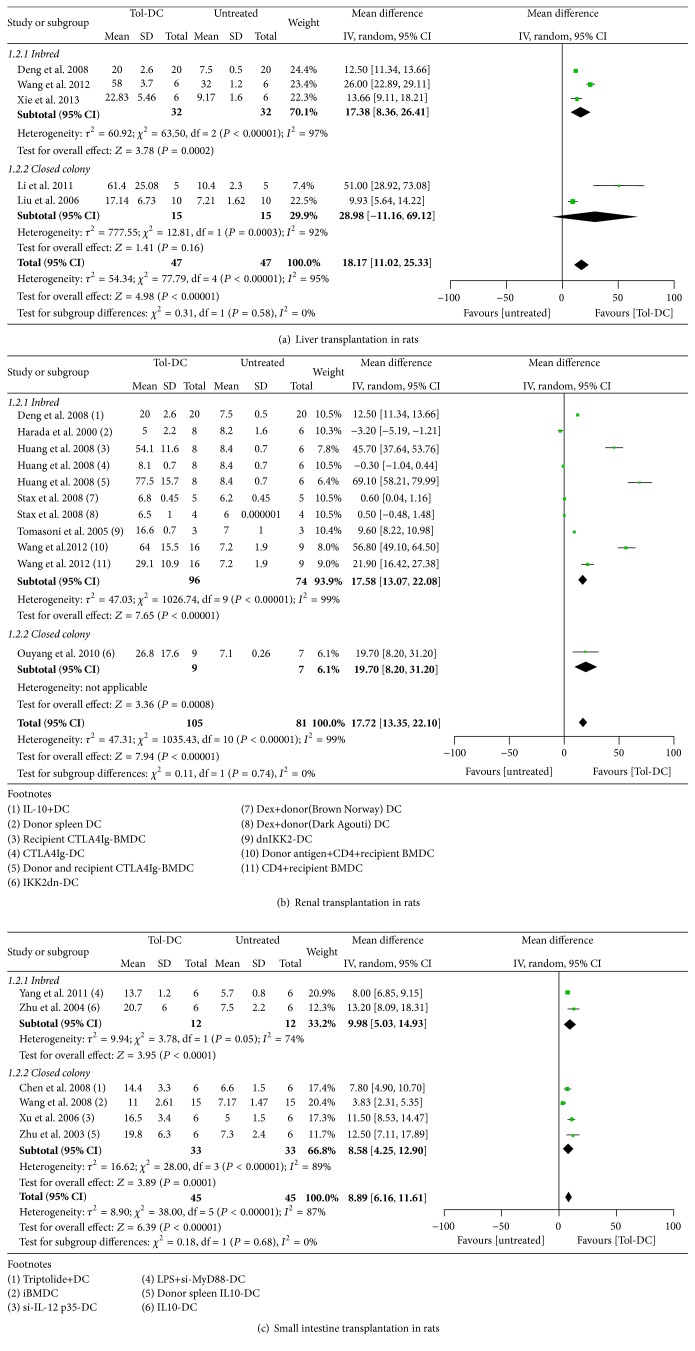
Mean difference (95% confidence intervals) for subgroups of inbred and closed colony rats for allografts survival in liver (a), renal (b), and small intestine (c) transplantation models. All mice and rats in other models are inbred.

All of the mice used in the included studies were inbred strains, while the rats used in the liver, kidney, and small intestine studies included both inbred strains and closed colony randomly bred animals. A subgroup analysis of the inbred strains and closed colonies revealed similarly prolonged survival times in the kidney and small intestine transplantation models, and the merged results were identical. In the liver transplantation model, despite negative merged result (28.98 days, −11.16 to 69.12) in closed colony, we tended to take it as positive because of positive results of both included studies, then identical to inbred strains (Figure 6). Therefore, no heterogeneity in allograft survival was observed in the inbred strains and closed colony animals.

## 4. Discussion

The current overview included a total of 61 articles (47 studies from six systematic reviews and 14 primary studies) dissecting the efficacy of adoptive transfusion of Tol-DCs with or without IS drugs in promoting the survival of heart, liver, kidney, small intestine, skin, and islet allografts in animals. Tol-DC therapy prolonged allograft survival to varying extents in all except the islet transplantation models. Moreover, Tol-DC combined with IS drug therapy further prolonged the survival of heart, skin, and islet allografts in mice, but not of heart allografts in rats. Compared with IS drugs alone, Tol-DC + IS therapy extended islet allograft survival in rats but failed to influence the survival of skin, small intestine, and heart allografts in rats or of heart and skin allografts in mice (Table 3). Although three articles reported that Tol-DC + IS therapy had better outcomes than IS alone in the mouse islet, rat liver, and kidney transplantation models, the evidence was based on single sets of data and we therefore did not include the results in our discussion. In addition, we did not directly compare Tol-DC and IS single therapies because most of the IS drugs have long histories of clinical use, whereas Tol-DC therapy has not yet been standardized and protocol differences undoubtedly affected the outcomes of the studies included here [10–15]. Comparisons of outcomes with single versus combination Tol-DC and IS therapy suggest that IS drugs have advantages over Tol-DCs. Our meta-analysis also shows that the efficacy of Tol-DC and Tol-DC + IS therapy varied with the transplantation model in both mice and rats, presumably reflecting the diversity of immune environments, organ-specific responses, and therapeutic protocols. Indeed, the severity and acuteness of rejection have been reported to vary for different grafts within a single organism, indicating organ-specific immune responses [75]. Moreover, in one transplantation model, the outcomes were different in rats and mice, suggesting species-specific responses to Tol-DC therapy.

### 4.1. Limitations

The following limitations of this overview are noted. (1) The studies used different strains of rats and mice. Gene expression in primary immunocytes varies greatly across inbred mouse strains, suggesting that the same Tol-DC therapy may have variable efficacy in different strains [76]. (2) The Tol-DC modification protocols and organ donor/recipient strain derivation differed among the studies. Thus, Tol-DCs with the same modification gave different outcomes depending on the transplanted organ and the donor/recipient combinations [54, 74, 77]. Additionally, different gene modifications, drugs, cytokines, and culture media can induce Tol-DCs with immature, mature, or semimature phenotypes. Immature DCs are conventionally considered to be tolerogenic and mature DCs to be immunogenic. However, immature Tol-DCs are not always superior to mature Tol-DCs in terms of allograft survival [38, 78]. (3) The studies differed in Tol-DC injection time, route, dose, and frequency. These factors influence Tol-DC efficacy to variable extents [37, 40, 65], presumably by affecting the distribution, maintenance, and homing of Tol-DCs [79, 80]. (4) The type of IS drug and the dose, time, and frequency of drug injection also differed considerably among the studies. (5) The statistical heterogeneity of the meta-analysis was very large for all transplantation models.

### 4.2. Clinical and Preclinical Implications

Our meta-analysis suggests that infusion of Tol-DCs alone is able to promote survival of allografts. However, there are currently no standardized protocols for the modification or application of Tol-DCs. To date, clinical experience with Tol-DCs is limited to a phase I trial of autologous Tol-DCs for type I diabetes, rheumatoid arthritis and refractory Crohn's disease, and an ongoing safety trial of autologous Tol-DCs for kidney transplantation [4–7]. Nevertheless, our results indicate that a number of problems must be solved before Tol-DC therapy successfully moves from bench to bedside. For instance, standardized protocols must be established for the modification and dose of Tol-DCs; the time, frequency, and route of injection; and the type of IS drugs to be administered in combination. Considering that Tol-DC efficacy may be organ-specific, the therapeutic protocol may also need to be tailored to the transplanted organ. Although Tol-DC therapy did not give better outcomes than IS therapy or show synergy with IS drugs, Tol-DC therapy does have advantages over drug therapy. First, Tol-DCs are generally infused before transplantation, and since they exert their effect at the very earliest stages of the immune response, they are very likely to inhibit hyperacute rejection. Second, Tol-DCs are likely to induce tolerance or prolong allograft survival without impairing the recipient's immune defense against other antigens. Third, the studies included in this overview did not administer IS drugs continuously. Therefore, Tol-DC therapy may allow IS drug use to be reduced, thus decreasing their toxicity and improving the recipients' quality of life, which is particularly important for IS-sensitive and IS-tolerant recipients. Notably, tacrolimus has been reported to inhibit the functions of Tol-DCs in mice [81], suggesting that further preclinical studies of Tol-DC and drug combinations are needed.

Our results indicate that Tol-DC efficacy may be species-specific, suggesting that studies in primates will be more clinically relevant. A kidney transplantation model has been successfully established in rhesus macaques and infusion of donor-derived Tol-DCs in combination with IS prolonged allograft survival [82]. This nonhuman primate model will help to translate research findings from animals to the clinic.

## Figures and Tables

**Table 1:** Characteristics of included systematic reviews.

Included reviews	Tx models	Animal models		Data synthesis	Included studies	Excluded studies	Potential new studies	Final included studies
Wu et al. 2012 [10]	Heart	Mice	Included		44	28	2	
		Rats	Not included		0	0	6	
		*Total*		Description	*44*	*28*	*8*	*24*

Sun et al. 2012 [11]	Islet	Mice	Included		9	7	3	
		Rats	Included		4	1	0	
		*Total*		Description	*13*	*8*	*3*	*8*

Xia et al. 2014 [12]	Liver	Mice	Not reported		0	0	0	
		Rats	Included		7	2	0	
		*Total*		Description	*7*	*2*	*0*	*5*

Xia et al. 2013 [13]	Renal	Mice	Included		5	5	0	
		Rats	Included		11	4	0	
		*Total*		Description	*16*	*9*	*0*	*7*

Zhou et al. 2013 [14]	Skin	Mice	Included		21	15	3	
		Rats	Not included		0	0	0	
		*Total*		Description	*21*	*15*	*3*	*9*

Sun et al. 2013 [15]	Small intestine	Mice	Not reported		0	0	0	
		Rats	Included		11	3	0	
		*Total*		Description & meta-analysis	*11*	*3*	*0*	*8*

*Total*	*5*				*112*	*65*	*14*	*61*

**Table 2:** Methodological quality assessment of systematic review.

SR	Model	Methodological quality assessment of the included systematic reviews, AMSTAR items											
		1	2	3	4	5	6	7	8	9	10	11	Rating
Sun et al. [15]	Small intestine	Yes	Yes	Yes	No	No	Yes	Yes	Yes	No	Yes	Yes	8
Zhou et al. [14]	Skin	Yes	Yes	Yes	No	No	Yes	Yes	Yes	N/A	No	Yes	7
Sun et al. [11]	Islet	Yes	Yes	Yes	No	No	Yes	Yes	Yes	N/A	No	Yes	7
Wu et al. [10]	Heart	Yes	Yes	Yes	No	No	Yes	Yes	No	N/A	No	Yes	6
Xia et al. [13]	Renal	Yes	No	Yes	No	No	Yes	Yes	Yes	N/A	No	Yes	6
Xia et al. [12]	Liver	Yes	No	Yes	No	No	Yes	Yes	No	N/A	No	Yes	5

Total		6	4	6	0	0	6	6	4	0	1	6	
%		100%	67%	100%	0%	0%	100%	100%	67%	0%	17%	100%	

N/A: not applicable. There are 11 items in total, “Yes” making 1 score and “No” or “N/A” 0.

**Table 3:** Effects of Tol-DC alone and Tol-DC in combination with IS on allografts survival. Values are mean difference (95% confidence intervals).

Transplantation models	Animal models	Tol-DC versus untreated	*Q* statistic	*I* ^2^ index	Tol-DC + IS versus Tol-DC	*Q* statistic	*I* ^2^ index	Tol-DC + IS versus IS	*Q* statistic	*I* ^2^ index
Liver Tx	Rats	18.17 (11.02 to 25.33)	*P* < 0.00001	High (95%)	NA^*∗*^	NA	NA	NA^*∗*^	NA	NA

Renal Tx	Rats	17.72 (13.35 to 22.10)	*P* < 0.00001	High (99%)	NA^*∗*^	NA	NA	NA^*∗*^	NA	NA

Heart Tx	Rats	14.21 (6.11 to 22.31)	*P* < 0.00001	High (100%)	*60.21 (−43.78 to 164.20)*	*P* < 0.00001	High (100%)	57.56 (−59.15 to 174.27)	*P* < 0.00001	High (100%)
	Mice	11.61 (7.73 to 15.49)	*P* < 0.00001	High (99%)	5.05 (1.53 to 8.57)	*P* < 0.00001	High (91%)	1.72 (−3.67 to 7.10)	*P* < 0.00001	High (93%)
	Total	12.78 (8.30 to 17.26)	*P* < 0.00001	High (100%)	31.47 (−33.22 to 96.16)	*P* < 0.00001	High (100%)	34.67 (−24.09 to 93.42)	*P* < 0.00001	High (100%)

Small intestine Tx	Rats	8.89 (6.16 to 11.61)	*P* < 0.00001	High (87%)	—	—	—	8.97 (−3.75 to 21.07)	*P* < 0.00001	High (99%)

Islet Tx	Rats	*7.28 (−2.91 to 17.46)*	*P* < 0.00001	High (98%)	137.49 (96.59 to 178.40)	*P* = 0.75	Low (0%)	*177.83 (160.05 to 195.62)*	*P* = 0.22	Low (33%)
	Mice	6.81 (2.97 to 10.64)	*P* = 0.01	High (70%)	NA^*∗*^	NA	NA	NA^*∗*^	NA	NA
	Total	7.70 (1.49 to 13.92)	*P* < 0.00001	High (97%)	108.21 (46.57 to 169.86)	*P* = 0.0009	High (82%)	133.26 (34.64 to 231.88)	*P* < 0.00001	High (100%)

Skin Tx	Mice	5.45 (2.31 to 8.59)	*P* < 0.00001	High (94.7%)	3.84 (3.40 to 4.29)	*p* = 0.22	Low (34%)	0.45 (0.00 to 0.89)	*P* = 0.22	Low (35%)
	Rats	—	—	—	—	—	—	7.15 (−3.84 to 18.13)	*P* < 0.00001	High (93%)
	Total	—	—	—	—	—	—	4.03 (−1.60 to 9.65)	*P* < 0.00001	High (92%)

—: no data. NA: not applicable. ^*∗*^Only one set of data in the subgroup.
